# From Immunologically Archaic to Neoteric Glycovaccines

**DOI:** 10.3390/vaccines5010004

**Published:** 2017-01-27

**Authors:** Marco Cavallari, Gennaro De Libero

**Affiliations:** 1 BIOSS Centre for Biological Signalling Studies, University of Freiburg, Schaenzlestrasse 18, 79104 Freiburg, Germany; 2 Experimental Immunology, Department of Biomedicine, University of Basel, Hebelstrasse 20, 4031 Basel, Switzerland

**Keywords:** vaccine, polysaccharide, serotype, conjugate, T cell help, immunosenescence

## Abstract

Polysaccharides (PS) are present in the outermost surface of bacteria and readily come in contact with immune cells. They interact with specific antibodies, which in turn confer protection from infections. Vaccines with PS from pneumococci, meningococci, *Haemophilus influenzae* type b, and *Salmonella typhi* may be protective, although with the important constraint of failing to generate permanent immunological memory. This limitation has in part been circumvented by conjugating glycovaccines to proteins that stimulate T helper cells and facilitate the establishment of immunological memory. Currently, protection evoked by conjugated PS vaccines lasts for a few years. The same approach failed with PS from staphylococci, *Streptococcus agalactiae*, and *Klebsiella*. All those germs cause severe infections in humans and often develop resistance to antibiotic therapy. Thereby, prevention is of increasing importance to better control outbreaks. As only 23 of more than 90 pneumococcal serotypes and 4 of 13 clinically relevant *Neisseria meningitidis* serogroups are covered by available vaccines there is still tremendous clinical need for PS vaccines. This review focuses on glycovaccines and the immunological mechanisms for their success or failure. We discuss recent advances that may facilitate generation of high affinity anti-PS antibodies and confer specific immunity and long-lasting protection.

## 1. Introduction

Pneumonia, meningitis, and bacteraemia remain major causes of death despite a century of polysaccharides (PS) vaccines and a decade short of reaching the century for conjugate vaccines [1,2]. Historically, vaccines were first developed as inactivated or attenuated whole-cell preparations. More recently, advances in biochemical methods led to the utilization of individual cell components in immunization, including PS, proteins, and DNA, whereas the use of glycolipids—except for adjuvants [3]—was neglected. The discovery of the relevance of T cell help [4] in the generation of antibody responses led to the preparation of conjugate vaccines including PS [5] and proteins. These vaccines were made of PS and proteins derived from different microorganisms, which in some instances diminished the great potential of these combinations. Reverse vaccinology based on next generation proteomic and gene sequencing closed the circle by designing highly complex multi-component vaccines that start to resemble whole-cell vaccines [6]. In some instances, the use of whole bacterial cells is returning, with interesting results [7]. Furthermore, recent advances in basic immunology are just beginning to uncover the important molecular aspects of host–pathogen interactions and the immunological principles that govern susceptibility to infection and protection of the host. Understanding these mechanisms is essential to replacing empirical vaccinology with rational design of vaccines [8].

## 2. Pure Polysaccharide Vaccines

**Figure 1:**
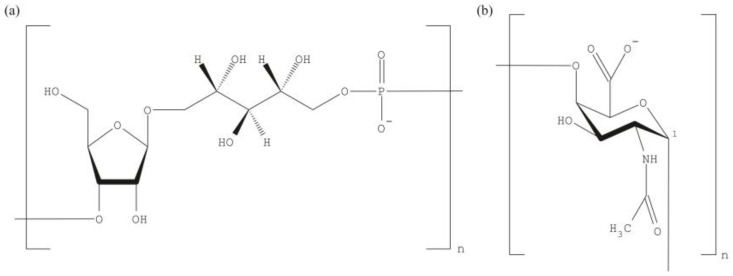
Repeating polysaccharide units of bacterial capsules. (**a**) The polyribosylribitol phosphate (PRP) repeating unit of *Haemophilus influenzae* type b (Hib) consists of two riboses (one reduced to ribitol) linked to a phosphate; (**b**) The capsular Vi antigen of *S. typhi* is built from a single sugar: *N*-acetyl galactosaminouronate. The monomeric Vi repeating units are α-1-4 linked.

The abundance of monosaccharides and the combinatorial diversity in PS are two reasons that justify why glycovaccines of polyvalent nature, i.e., containing two or more strains/serotypes of the same antigen, require extensive clinical validation. Usually, each vaccine component is individually tested before the complex mixture enters any trial. In the simplest case, a single saccharide is the building block for the PS. For instance, the polyribosylribitol phosphate (PRP) is the repeating unit in the *Haemophilus influenzae* type b (Hib) capsule (Figure 1a) while the Vi antigen of *Salmonella typhi* is composed of α-1-4 linked monomers of *N*-acetyl galactosaminouronate (Figure 1b). Pneumococcal capsules are more complex and consist of di- to octa-saccharide repeating units; reviewed in [9]. The synthetic preparation of units that exactly mimic the natural ones represents difficult challenges. Complications in vaccine formulation from PS arise due to contaminants [10], mostly teichoic acid, and labile saccharide modifications such as *O*-acetyls. Teichoic acid is itself immunogenic [11] and so might divert the immune response from the critical PS epitope. The loss of various, labile *O*-acetylations might destroy the immunogenicity of a PS and in turn lead to an ineffective vaccine. Indeed, some pneumococcal serotypes differ in just one *O*-acetylation [9]. The over 90 serotypes were originally defined by the Quellung reaction that is based on antibodies raised against the capsules. Thus, we can conclude that our immune system is able to distinguish epitope differences as small as one sole *O*-acetyl group. 

A consideration on PS as antibody targets is the size of the antibody footprint interacting with the antigen. Even though antibodies may discriminate very small molecules (around one square nanometer surface), the usual antibody footprint is five to twenty square nanometers and covers an area in the range of a tetra-up to a deca-saccharide [12]. These structural constraints indicate that the smaller a PS the fewer potential immune epitopes it contains. On the other hand, larger PS might form helical, condensed, or other complex structures that conceal the antibody epitope [13]. Furthermore, the more similar they are to human PS—such as group B meningococcal PS [14]—the weaker the immune response they generate because of tolerance to self. 

Another issue related to PS immunogenicity is the number of repeats of the PS repeating unit. These are important because polyvalent PS trigger B cells independently of dedicated presentation or of help provided by other cell types [15,16,17]. As the B cell response occurring in the absence of T cell help may be very different from that elicited in the presence of T cells providing cognate help, this issue has great impact on the design of synthetic vaccines. Furthermore, the presence of repetitive structures increases the avidity of antibody-antigen binding, thus facilitating the activation of B cells even when they express low affinity antibodies.

Current improvements in PS synthesis [18] and glyconanotechnology [19] might soon allow the production of synthetic PS vaccines [20]. Only few nanomaterials have been described as scaffolds for PS vaccines, among them gold glyconanoparticles. Inert and non-toxic in nature [21], they can be used to assemble PS in defined ratios (alone or together with conjugates and adjuvants) but they seem poorly immunogenic [19]. Such vaccine formulations have to be thoroughly tested to exclude adverse events especially considering size effects of nanoparticles [22,23] and molecular resemblance of foreign PS to human ones, e.g., group B meningococcal PS [14]. Such mimetics might in the worst case provoke the converse of the intended immune response, namely tolerance. Such a mechanism was reported for PS binding to the sialic acid-binding immunoglobulin-type lectin (Siglec) CD22 [24,25], a regulator of B cell tolerance [26,27,28]. Liposomal nanoparticles that display CD22-recognized PS together with antigens on their surface stalled B cell responses by apoptosis.

To date, many vaccine formulations still rely on the purification of PS from cultured bacteria. This type of manufacture requires batch to batch quality controls that grow exponentially from the quadrivalent meningococcal (MPSV-4) to the 23-valent pneumococcal (PPV23) vaccine. Therefore, covering all 90 plus pneumococcal serotypes will never be cost-effective in contrast to lower valency vaccines as predicted by current health economic models [29].

Pure PS vaccines entered the market in the 1970s (MPSV-4, PPV14) and 1980s (Vi, PPV23, and Hib). The 23-valent pneumococcal PS vaccine (PPV23) replaced the 14-valent PPV14. After several decades, successor products for three of the other four vaccines are still in use; only the Hib PS has been withdrawn. Only PS-based vaccines were substituted by those in which PS is conjugated to different carrier proteins. Conjugate vaccines have been introduced for MPSV-4, Vi, and PPV23 albeit the valency of the pneumococcal vaccine is reduced (7- to 13-valent) in the conjugate versions.

Invasive *Salmonella* globally represents 5 to 6 serovars that could be covered by a polyvalent vaccine [30]. Other serovars such as *S. paratyphi A*, *B*, and non-typhoidal *Salmonella enterica* (NTS) cause enteric fever but, with rare exceptions, lack a PS capsule. Their surface O–PS of lipopolysaccharide (OPS) could be used to induce protection [30]. Combination of Vi and OPS in one vaccine might be protective against encapsulated and capsule-free *Salmonella* if neither component is immunodominant because Vi hides bacteria from recognition by OPS- and non-Vi specific antibodies [31].

Some infectious agents can be either invasive or non-invasive. Non-invasive pneumococcal infections outnumber invasive ones 3 to 1 [32]. The virulence contribution of the capsule is clearly documented but it is not the sole factor to affect community-acquired pneumonia (CAP) and invasive pneumococcal disease (IPD) risks [33]. During non-invasive infections pneumococci exploit different types of strategies to escape immune surveillance. A panoply of proteins are involved in immune evasion and they represent potential vaccine candidates [34]. In some instances, these strategies allow pneumococci hiding inside erythrocytes [35]. Thereby, they are protected from major immune attacks by neutrophils, antibody-dependent cell-mediated cytotoxicity (ADCC), complement-dependent cytotoxicity (CDC), hydrogen peroxide and also antibiotics. Because erythrocytes do not express major histocompatibility complex (MHC) molecules, intracellular pneumococci are protected from T cells too. Such tremendous pathogenic potential, justify the rather short-term (maximally several years) protection of PS vaccines. In addition, PS vaccines promote efficient protection only in adults—not in infants, the elderly and immunocompromised individuals—and fail in booster immunizations [33]. Thereby, this unique type of infection requires novel approaches to detect and eliminate hiding pneumococci.

## 3. Protein Conjugate Vaccines

When B cells are stimulated by antigen and concomitantly receive cognate T cell help they activate unique cellular programs that include proliferation, maturation, differentiation into plasma cells and they also reactivate molecular mechanisms of immunoglobulin gene diversification such as somatic hypermutation (SHM) and class-switch recombination (CSR) [4,36]. These processes are much less frequent when B cells are activated by polyvalent antigens with many repetitive units and in the absence of T cell help. This integrated and highly regulated program is the explanation why PS conjugated with protein antigens that stimulate specific T cells give rise to antibodies with high affinity, induce long lasting B cell memory and reduced B cell tolerance. Because of the involvement of T cells, this type of response is defined as thymus-dependent (TD), in opposition to the response defined as thymus-independent (TI) observed when non-conjugated polyvalent PS are used during immunization.

Among the most frequently used TD antigens linked to PS are tetanus toxoid (TT), diphtheria toxoid (DT), cross-reacting material (CRM197) of diphtheria toxin, the outer membrane protein complex (OMPC) of *Neisseria meningitidis* serogroup B strain B11, and the outer membrane protein D (PD) derived from non-typeable *H. influenzae*. TT and DT were discovered on the verge of the twentieth century when Gaston Ramon inactivated the respective toxins employing formalin. CRM197 and DT are structurally highly similar but under reducing conditions the nicotinamide adenine dinucleotide glycohydrolase activity of CRM197 is eliminated upon introduction of a G52E mutation that renders the active-site loop more flexible [37]. Two effects have to be balanced if such carrier proteins are used: (1) maintenance of an effective T cell help; and (2) immune interference caused by a carrier that diverts the immune system from the relevant target [38]. Diversion of specific immunity might happen to a pre-existing immunity to PS, in which PS-specific B cells might not receive adequate help [39]. These adverse effects remain a serious reason of vaccine failure, as conjugate vaccines require boost immunizations that might divert, rather than increase the specific immunity [40,41].

Despite these important limitations, several glycovaccines are available on the market (Table 1). 

Both PS purification from bacterial cultures and protein conjugation can affect PS immunogenicity. Initially, the anomeric hydroxyl group at the reducing end of saccharides was the preferred coupling site [42,43]. In many instances this terminal conjugation preserves the PS antigenic epitopes. However, during conjugation the protein part might also become altered, leading to novel immunogenic proteins that can induce allergic responses as reported for CRM197 [44] or the IgE-dependent sensitization to PS [45]. Such allergic reactions might be avoided by more modern conjugates [46] that are capable of suppressing allergy [47] and can be linked to PS on specific sites [46,48,49,50].

In comparison to the 23-valent pure PS vaccine the conjugate vaccines only include a selection of serotypes reaching 13 PS in total and with the prospect of a 15-valent version [51]. Composition of PCV7 and PCV13 were based on the serotype prevalence in the Western world; a fact that is reflected by the poor coverage in East and Southeast Asia e.g., 60% of the infective serotypes in China [52]. The production costs might prevent the use of conjugate vaccines in developing countries where the emergence of antibiotic resistant bacteria occurs at high rate. On the evidence of the relation between vaccine serotypes and antibiotic resistance [53], supportive programs should be implemented. In Germany, the meningitis serotype distribution changed to non-PCV13 types and went along with a reduction in antibiotic resistant isolates. On the other hand, resistant pneumococci are increasingly isolated world-wide [54,55,56].

Although conjugate vaccines are unable to prevent colonization, the immune system removes the covered serotypes using various effector mechanisms including antibody-mediated, Th1, and Th17 immune responses [57,58,59,60]. The Th17 response deserves a special note because it could provide a serotype-independent defence mechanism involving B cells and innate interleukin (IL)-17 [61]. Furthermore, IL-17 producing CD4 T cells specific for one serotype were effectively de-colonizing another serotype not expressing the specific antigen [62,63]. Since this type of response is antibody-independent it will not prevent colonization but could be used to clear cohabitants. The authors also speculated that the escape from a vaccine eliciting a Th17 response might be slower than from a vaccine triggering a humoral response. The antigens they used represented pneumococcal lipoproteins and in part acted by activation of Toll-like receptor 2 [64]. Generally, Th17 has been described as an immune response evoked by intranasal immunizations [65]. However, a special immune cell type, the semi-invariant natural killer T (iNKT) cells, can be exploited to deviate the intranasal route from a Th17 response [66]. 

Conclusively, current vaccines offer protection against the bacterial strains they cover. However, they are incapable of preventing carriage by non-vaccine-covered serotypes [67].

## 4. Multiple Causes May Prevent the Efficacy of Bacterial Vaccines

Insufficient immunological knowledge probably explains why other vaccines remain very inefficient. For example, despite worldwide efforts, a vaccine for the clinically important *Staphylococcus aureus* has not made it to the market, yet. Basic research showed the importance of Th1 and Th17 responses in defence against staphylococci [68,69]. While Th17 is prominent in *S. aureus* clearance from skin and respiratory infections, Th1 responses protect from systemic infection. Recent findings showed that IL-17-secreting γδ T cells play a crucial role in protecting from skin-invading staphylococci. γδ T cells are a major population of T cells in humans [70] and although their number and activation state is increased during infections no vaccine harnesses them as yet.

The PS capsule of *S. aureus* helps this bacterium to resist the immune response and facilitates the establishment of infection. The biosynthetic pathway that builds the capsule components has been intensely investigated in several studies [9,71,72,73,74,75] which, however, have not provided useful clues to combat these infections. The two major disease-causing serotypes 5 and 8 produce capsular PS that do not induce specific immunity even when conjugated to TT [76]. Only the addition of several staphylococcal proteins leads to a protective immunity, thus raising the question of whether the T cell response elicited by a vaccine needs to be pathogen-specific [77], at least for certain pathogens. A pure protein vaccine against *S. aureus* is not available despite a multitude of studies [68,78,79,80]. Probably, the combination of infection proteomics [81] with PS biosynthesis will lead to successful staphylococcal vaccines. In line with this hypothesis, infection of the mouse gastrointestinal tract by *S. aureus* necessitates the presence of both intact teichoic acid, the capsular PS, and surface proteins [82]. In addition, others have shown that the staphylococcal capsule acts in concert with proteins to shield the bacterium from the host [83].

A limitation in vaccine development is the lack of animal models that closely mimic human disease and are translatable in terms of their immune response [84,85,86,87,88]. For instance, even immunocompromised mice are resistant to persistent infection with *Staphylococcus epidermidis* [89]. Therefore, one has to carefully weigh the advantages and disadvantages of a certain animal model for vaccine testing. An experimental human pneumococcal colonization model [90,91] has been successfully used to test a pneumococcal vaccine [92] and similar human models for other bacteria might overcome the limitations of animal models. A possible solution to investigating new vaccines would be testing in immunocompetent healthy adults. However, the same approach might be problematic in infants, elderly, and immunosuppressed or immunocompromised persons, who are the most frequent subjects of vaccination.

## 5. The Immune System Comes of Age and Senesces

In addition to immunosuppressed and -compromised people, the elderly and infants are high risk groups. The reasons are multiple and also include an immature, or a senescing immune system. Vaccination of immunologically healthy individuals who are taking immunosuppressive medication might be tackled by appropriate timing in the administration of both the vaccine and the immunosuppressant. Autoimmune disorders may, or may not compromise the efficacy of vaccination depending on the type of autoimmune disease and of affected cell population—if B and T cells are normal then immunizations are likely to be successful. Moreover, if the disease is treatable—e.g., by replacement therapy of the missing factor(s)—vaccination of immunodeficient individuals can be efficacious. Immature or senescing immune cells [93] occur in children and old people, respectively, with a clear impact on vaccination [94]. In developed countries, at least, the demographic pyramid is more and more inverted, which creates a huge impact on public health and vaccination [95,96]. Usually, the spread of a disease is faster among children with the same life-style, while both infants and the elderly may be identically endangered. Over the last decades pensioners have become more active and adventurous and have an extended lifespan. They travel the world more often, which brings them in contact with pathogens not endemic in their home countries. At the same time, they might become carriers of pathogens that are new to their travel destinations. This often under evaluated aspect poses a major threat to indigenous populations [97,98] as shown by the disastrous consequences of importing a new pathogen during the colonization of the new world shortly after its discovery. Another concern is the efficacy of travel vaccines in the elderly [99]—a matter that remains poorly studied. The immune system is probably less efficient in mounting a fully protective response to such neoantigens. Moreover, in elderly more frequent side effects may be induced by attenuated live vaccines, such as the yellow fever vaccine, because the virus present in the vaccine is still infectious.

In summary, the effectiveness of an immunization correlates with immunosenescence [100], but also with gender [101]. With age the adaptive immune system changes, since memory T cells predominate and thymic output of naïve T cells decreases [102,103,104]. Furthermore, precocious aging of the immune system may occur in some common metabolic syndromes, which are becoming very diffuse in western countries. For example, accelerated ageing of the thymus [105] and decay in T cell egress from thymus occurs in obese people [106]. Another age-associated change is the ratio between regulatory (Treg) and helper (Th) T cells. While the age-related increase in Treg cells is beneficial in the context of autoimmunity [107], the imbalanced Treg vs. Th cells might profoundly alter the response to vaccine [108]. Immunization in advanced age leads to lower conversion and seroprotection (generation of vaccine-specific antibodies and increase of protective ones, respectively) either after primary or booster vaccination [99,109]. Whereas B cells are generated throughout life [110], there are age-related changes in the number of recent bone marrow emigrants and the humoral repertoire [111,112]. These changes seem to correlate with alterations in E2A expression and regulation, a transcription factor controlling both the B-cell precursor and mature B-cell developmental stages [113]. E2A has also a major role in the transcription of activation-induced cytidine deaminase (AID), the key enzyme for B cells to enter CSR and SHM. Hence, E2A age-dependent changes critically influence the effector functions and affinities of antibodies e.g., by lowering the AID levels and thus impeding CSR and SHM of B cells. Other changes seem associated with the presence of low expression of λ-5 (a protein of the pre-B cell receptor surrogate light chain) leading to reduced expression of the pre-B cell receptor, which is important for pre-B cell expansion and the selection of the immunoglobulin heavy chain variable domain [114]. These alterations cause the generation of a small B cell repertoire and poor humoral immunity. 

In addition to the consequences on T and B cells, aging also affects haematopoiesis with a shift towards increased generation of cells of the myeloid lineage [115,116]. This bias may be caused by the selective limitation of lymphoid hematopoietic stem cells to self-renew in response to DNA damage or telomere dysfunction [117]—both events accumulating with age. A crucial factor that keeps the myeloid lineage in check is GADD34. This protein when expressed in myeloid cells inhibits granulocyte-colony stimulating factor (G-CSF) receptor signalling and blocks stem cell and/or precursor proliferation and differentiation [118]. All together these changes have a negative impact on the immune response to vaccination and thus reduce the efficacy of vaccines in elderly.

Despite the profound alterations in haematopoiesis, vaccination can also be successful in aged individuals [119]. For example, vaccination with the polysaccharide PCV7 outperformed PPV23. Both vaccines were safe and induced serotype-specific opsonizing antibodies. Few studies are reported for such age groups and even fewer compared the extremes of age in systemic approaches [120]. This lack of information may have important effects on public health. Indeed, lack of adequate experimental data is delaying the licensing of PC-based vaccines in elderly. Indeed, the *Neisseria meningitides* conjugate vaccines have not been approved yet and only the MPSV-4 vaccine is available, probably the less effective variant.

Clinical trials to evaluate the effects of child immunization are readily terminated because it is easy to recruit the appropriate cohorts [121]. Furthermore, governmental health programs often target the youngest to prevent disease at the earliest age possible. In this age group an important aspect is that PS vaccines may fail due to the infants’ immunojuvenility, characterized by a naïve, developing immune system lacking immunological memory [122]. Development of the immune system starts at specific locations in the foetus [123,124] and ripens rapidly during the first 3 years of life. During this time, only conjugate vaccines [41] induce seroprotection. In most cases, such protection is assessed by the antibody titres pre- and post-immunization. This type of evaluation might not be sufficient to confirm the protective outcome of vaccination, as the quality of the generated antibodies is the most relevant type of response [99]. The titre reflects the occurrence and the quantity of vaccine-specific antibodies whereas the quality is more appropriately mirrored by their affinity and repertoire [125,126,127,128,129].

## 6. Herd Immunity

Modelling often implies that preferential vaccination of high risk groups is beneficial for the whole group of people and reduces the required vaccination rate in the total population [130]. Such models ignore that fact that, due to the above mentioned immunological constraints, the high risk groups are often less responsive to a given vaccine. Furthermore, vaccines are never 100% effective at the population level due to polymorphisms of the immune system and/or immune deficiencies and logistically it is impossible to cover 100% of mankind. These considerations suggest that to protect the whole population, herd immunity has to be achieved [131]. Immunity of a population (herd) is secured if the pathogen is hindered from spreading—a quite old and still important immunological concept [132]. Usually, infection of a susceptible individual by a carrier is estimated unlikely for population immunization rates above 85%. Unfortunately, these rates are not easily attained nowadays. Paradoxically, this might even be a consequence of the unique success of vaccines through history because we are confronted with some infectious diseases much less frequently than in pre-vaccination eras. A fact that might sharply change in the future is the re-emergence of infectious diseases incorrectly believed extinct or eradicated. Measles represents a first example. 

Mathematical modelling of herd immunity mainly neglect current immunological flaws of vaccines but can try to evaluate and integrate societal sentiments about vaccination [130,133,134,135]. Social and religious cluster analysis also reveals that clusters of susceptible persons are likely to occur. Therefore, social behaviour and parenting will impact on the speed of disease spread [136]. Furthermore, neglecting the vaccination rate of the elderly will prohibit herd immunity altogether [137]. If the treatment of the disease fails or there is none, high vaccination rates become critical to prevent a disease outbreak [138]. In this case, even poorly contagious pathogens will require immunization rates above 40% [138]. Especially with nosocomial multi-resistant strains on the rise, herd immunity might be crucial to avoid disease outbreak and the spread of antibiotic resistances between germs [139,140]. Political decision makers have sufficient tools at their command to judge a vaccine [29,133] but even in the case of a successful evaluation, the vaccination rates necessary to establish herd immunity will remain difficult to achieve and impossible to impose to entire population.

The gold standard in effectiveness are live attenuated viral vaccines that in many instances confer life-long immunity after a single shot. Attenuated bacterial vaccines are inexistent to date and PS as well as conjugate vaccines still suffer immunological knowledge gaps [40,141]. This fact is reflected in many studies that reported conflicting results concerning the protection of adults after pneumococcal conjugate vaccine introduction for infants. In Brazil, PCV10 protected the vaccinated infants from IPD whereas older individuals suffered from increased IPD rates [142]. In the UK, on the other hand, CAP declined in adults after introduction of PCV7 (2008) and PCV13 (2010) indicating the establishment of a potential herd immunity [143]. In Spain, the vaccination of children reduced the mortality due to IPD in adults but the case fatality rate in over 65 years old patients remained unchanged [144]. In elderly, non-PCV serotypes were the main cause of death. The apparent incongruence of those studies highlights that bacterial (pneumococcal) carriage is distinct in different geographical regions and also differs with the age of the carrier.

## 7. Bacterial Genomics and Proteomics (Sero- and Resistance-Typing)

The tedious determination of bacterial serotypes is being overcome by the recent advancement of typing methods [145,146]. Especially for pneumococci, encapsulated by more than 90 PS varieties, fast and specific sero- as well as resistance typing is critical to deciding on the treatment of the infection. *Streptococcus pneumoniae*, responsible for the majority of CAP, has a very different prevalence in carriage amongst infants and adults: it might represent up to 60% in children aged 2 to 3 years but less than 10% in grown-ups whereas *S. aureus* is dominant around 10 years of age [55,147]. World-wide, a substantial percentage of pneumococcal, staphylococcal and other bacterial isolates are resistant to antibiotics (WHO). This resistance makes the generation of more effective vaccines mandatory.

The major challenge is to perform next generation sequencing (NGS) and advanced proteomic methods on bacterial samples without culturing. Obviously, the internal life-cycle of a bacterium and the environment will directly influence its gene expression. NGS can be performed on limiting sample amounts and will tell the presence of known antibiotic resistance markers. In contrast, non-biased mass spectrometry requires larger quantities. Multiple reaction monitoring (MRM) mass spectrometry can be performed on smaller sample sizes but the target proteins have to be known and their fragmentation pattern defined. MRM allows the quantification of the sample in absolute terms. After many strains have been sequenced [148,149] and their proteome analysed [67], the time is ripe to assess the transition from bacterial colonization to infection. Such studies would for the first time address the time-dependent and host-influenced gene expression changes that occur when bacteria change from a commensal to an invasive and pathogenic state. Bacterial proteomics and NGS may also shed light on how cell wall biosynthesis is connected to changes in the capsular serotype.

## 8. Cell Wall Polysaccharides and Serotype Replacement

Most bacteria evolve rapidly and adapt quickly to their surroundings. Pneumococci are competent for natural transformation that allows them to acquire genetic information in the form of naked DNA from the environment [150]. This elaborate competence mechanism [151] involving fratricide plays a key role in the evolution of the PS capsules and serotype replacement [152]. Some of the competence genes for fratricide are active on closely related species such as *Streptococcus mitis* and *oralis*. However, the participation of other mechanisms to facilitate the integration of genetic information of related species into the pneumococcal genome is probably important. One possible approach to further investigate the involved genetic mechanisms may be offered by NGS on bacterial communities [148,149,153,154]. The transfer between pneumococci that leads to serotype replacement and transfer of antibiotic resistance is undeniable. More than 70% of the pneumococcal reference genome has recombined in at least one sample and on average 74 kilobases were exchanged [154]. Given the tremendous flexibility caused by transformation, replacement in carriage is unavoidable without complete vaccine coverage of all serotypes and should lead to anything but carriage of more pathogenic serotypes. A lack of effect was observed after the introduction of PCV13 in the US, where the number of meningitis cases in children remained unchanged [155]. It is noteworthy that serotype 19A still represented a substantial number of hospitalized cases even though it is included in the PCV13. This finding might suggest the occurrence of competition in multi-valent vaccines such as PCV13 [54,156]. Persistence of vaccine serotypes has been noted earlier [155,157] and it has been reported that persistence and serotype replacement may have a geographical component [158].

A major concern is the occupation of a possible colonization niche—after vaccination or antibiotic treatment—with a more rogue bacterium [159]. *S. aureus* is a candidate to take over pneumococcal carriage after successful vaccination [160]. With the caveat of using epitopes that are present in our microbiome as well, antigens shared between *S. pneumoniae* and *S. aureus* have been described but not used in a vaccination approach, yet [161].

## 9. Needs for Novel Bacterial Polysaccharide Vaccines

Many Gram-positive and Gram-negative bacteria produce capsular PS. Such PS usually come in many structural varieties and often uniquely define a certain bacterial strain. The generation of novel vaccines inducing protective immunity against these unique PS may provide new achievements in the field of PS vaccination. At least two species deserve special mentioning: *Streptococcus agalactiae* (GBS) *Klebsiella pneumoniae* and *Campylobacter jejuni*. 

GBS is a human commensal and is carried up to 30% of healthy individuals. It is the major cause of infection after childbirth and is responsible for many casualties among newborns [162,163,164]. Several reasons would suggest using vaccination and not antibiotic treatment for GBS eradication. Increasing GBS antibiotic resistance hampers the treatment of pregnant women. Furthermore, the eradication of all microbiota by antibiotic therapy may widely influence the immune system in several ways, including cell development, responsiveness to vaccination, immune regulation, and appearance of food allergies [165,166,167,168,169,170]. A GBS vaccine would contribute to eradication without such adverse effects on the immune system. 

In the case of infections with *K. pneumoniae* the escalation of multi-resistant strains is being observed, especially in hospital settings. This resistance is often combined with a high mortality [171,172,173], which demonstrates the compelling need for an effective *Klebsiella* vaccine.

Infections with *Campylobacter jejuni* represent another important burden that could be treated with efficient vaccination. *C. jejuni* infections have an estimated incidence rate of 0.3% to 1.5% [174]. They are the major cause of bacterial diarrhoea and no vaccine is available at present. Occurrence can reach 5% to 15% in travellers and post-campylobacteriosis complications can include irritable bowel syndrome, inflammatory bowel disease, arthritis, and a high percentage of foodborne illnesses. Importantly, *C. jejuni* infections are associated with insurgence of Guillain-Barré syndrome characterized by acute flaccid paralysis. This side effect is the consequence of shared sugar structures between gangliosides in nerve cells and lipooligosaccharides in bacteria. Antibodies cross-reactive to both types of sugars are responsible for the neurological symptoms. A vaccine targeting the *C. jejuni* PS [175] should avoid this type of cross-reactive immune response. 

## 10. Future Directions for Glycovaccines

Future PS vaccines will take great advantage by integrating latest immunological knowledge and exploiting modern biotechnology tools. 

**Figure 2:**
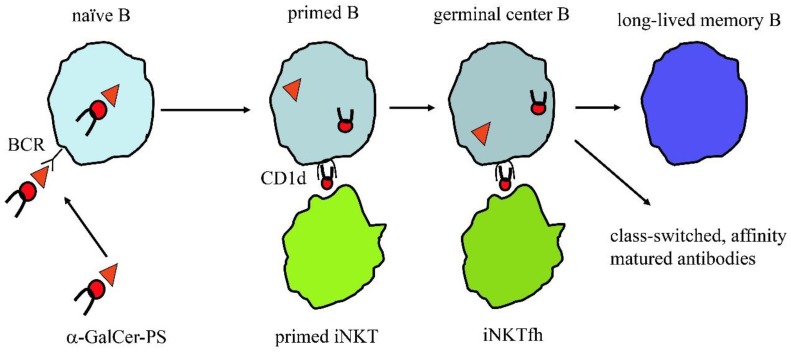
Polysaccharide coupled α-GalCer induces long-lived memory B cells and high-affinity, class-switched antibodies with the help of iNKT cells. A naïve B cell endocytoses the polysaccharide vaccine (α-GalCer-PS) via its B cell receptor (BCR). The vaccine is processed during its trafficking through the endolysosome and α-galactosylceramide (α-GalCer), an antigen stimulating all iNKT cells, is released from the complex. The free antigen is loaded onto CD1d molecules that also recycles within same endocytic compartment. The CD1d-α-GalCer complexes traffic to the plasma membrane and stimulate iNKT cells. Activated iNKT cells develop into follicular helper iNKT (iNKTfh) cells that participate in a germinal center reaction together with PS-specific B cells and contribute to generation of hypermutated, class-switched antibodies and establishment of immunological memory.

The latest studies on innate T cells, which are an abundant subpopulation of human T cells (about 5%–10% of circulating T cells), revealed new opportunities to generate PS vaccines of high efficacy. A recent study showed that *S. pneumoniae* PS coupled with synthetic glycolipid antigen stimulating iNKT cells induces high titres of antibodies that are class switched, of high affinity and specific for the PS used in the vaccination (Figure 2). Such a strategy also induced the generation of long-term memory B cells and plasma cells secreting PS-specific antibodies, which protected mice from a lethal infection [46]. This approach relies on two important concepts: the first is that B cells producing PS-specific antibodies preferentially internalize the conjugate vaccine and present the covalently-linked glycolipid antigen to iNKT cells. The second concept is that as iNKT cells are abundant and preactivated, they deliver immediate help to PS-specific B cells, without need of clonal expansion, nor of functional maturation. The added bonus of this approach is that iNKT cells are restricted by the non-polymorphic CD1d molecule that is expressed by B cells. Thus, this vaccine is predicted to work in all individuals, independently of their HLA haplotype.

Another recent observation was that most microbe-specific naïve CD4^+^ T cells produce memory cells during infection [176]. The identification of the antigen specificities of the memory T cells naturally generated during infection will be of great value to incorporate the appropriate antigens within vaccines to elicit the exact type of response occurring during infection. 

Importantly, the anatomical site of infection contributes to the type of immune response. A vaccine aimed at inducing protective humoral immunity in mucosae should also induce antibodies of the IgA isotype and T cells with the capacity to migrate into those tissues. These responses might be influenced by the type of vaccine delivery and the adjuvant used.

**Figure 3:**
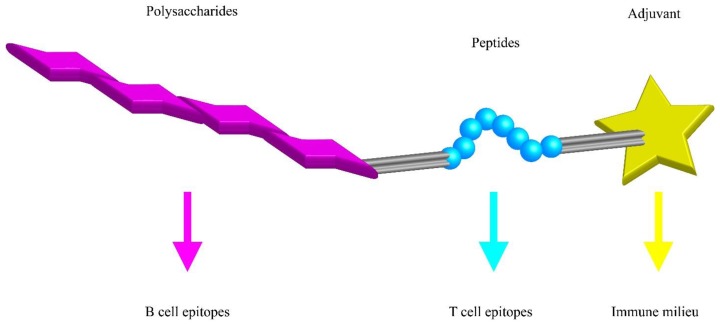
Synthetic polysaccharide vaccine building blocks. A minimal polysaccharide (PS) vaccine needs to provide the B and T cell epitope together with an adjuvant that determines the nature of the immune response. Peptides are used as example where classic T cells would help B cells.

The great improvements in biotechnology have also opened many new avenues to PS-based vaccines. Ideally, vaccines could be constructed from a set of building blocks each providing unique signals to immune cells. Multi-component vaccines might be made of a synthetic PS conjugated with a T cell antigen and one or more adjuvants controlling the pro- or anti-inflammatory type of the immune response (Figure 3). Furthermore, these vaccines might be used to influence the cytokine milieu at the place of the T-B interaction and provide the signal three for the B cell that steers the isotype switch in CSR.

Knowledge of the structures recognized by PS-specific antibodies is very important. The identification of the bacterial PS epitopes stimulating protective B cells could lead to the synthesis of PS analogs eliciting the same antibodies that interact with native PS on infecting bacteria. Such an approach has already been proven successful in several instances [177,178]. However, these examples applied to protein antigens and similar studies are still in their infancy with PS-specific antibodies.

Structural studies might also inspire other types of innovative and unconventional approaches to counteract bacterial immune evasion strategies. For example, staphylococcal proteases cleave the hinge region of IgG, thus reducing the protective potential of humoral immunity. By inducing autoantibodies against this region, it was possible to restore IgG immune functions that prevented bacterial colonization [179].

Attention has to be paid to circulating anti-foreign saccharide antibodies naturally present at high levels in human serum that arise from our diet or microbiome—e.g., anti-Neu5Gc and anti-Gal antibodies, respectively [180]. Both types of antibodies might be beneficial in a dose-dependent manner in anti-infection therapy. However, they might also promote cancer through chronic inflammation or hamper vaccination inducing immune diversion.

Other important areas for the development of glycovaccines are anti-fungal and anti-tumor vaccines. Presently, there are no anti-fungal vaccines although they might have great clinical impact. Hybrid vaccines combining glycoproteins containing known B and T cell epitopes and adjuvants might represent an appropriate approach. The main cause of candidiasis, *Candida albicans*, expresses the phosphomannan glycoprotein that is linked to β-mannan. Protective vaccination was achieved by phosphomannan disaccharide structures eliciting protective antibody responses in mice [12]. More impressively, this study described a hybrid vaccine composed of (1) a β-mannan trisaccharide; (2) a 14 amino acid peptide of the fructose-bisphosphate aldolase of *C. albicans*; and (3) TT that did not require any adjuvant [181]. Using this novel tripartite combined vaccine, high titre antibodies and specific T cell immunity were observed.

Similar hybrid vaccine approaches containing tumor-associated carbohydrate antigens (TACA) are under investigation [182]. TACA arise from the altered expression of glycosyltransferases in malignant cells and are often made of much shorter chains than carbohydrate chains generated in healthy individuals. This form of altered self is generally poorly immunogenic [180]. In addition, an increase of sialic acids on cancer glycoproteins and glycolipids help evade the killing by natural killer cells that receive an inhibitory signal via Siglec-7 [183]. Clever manipulation of immune responses could turn the tables on the evader by using the very same receptor to trigger immunity. Indeed, an adaptive CD1b-restricted T cell response was evoked by delivering mycobacterial antigens to human dendritic cells in a Siglec-7-dependent fashion [184].

The full picture of the interaction of the immune system with PS has just started to be unravelled. The possibilities for immune manipulation using PS seem very broad and will depend on deeper knowledge of the underlying molecular mechanisms regulating this type of recognition. Novel approaches in synthesis, conjugation, delivery and immuno-monitoring will greatly increase the value of glycovaccines and will tune their efficacy in protecting the population.

## Figures and Tables

**Table 1:** PS vaccines available on the market.

Vaccine	Date	Conjugate	FDA	EMA	PS
ActHIB	27 September 1996	TT	Sanofi Pasteur, S.A.		Hib (PRP)
Hexacima	17 April 2013	TT		Sanofi Pasteur S.A.	Hib (PRP)
Hexyon	17 April 2013	TT		Sanofi Pasteur MSD SNC	Hib (PRP)
Hiberix	19 August 2009	TT	GlaxoSmithKline Biologicals, S.A.		Hib (PRP)
Infanrix Hexa	23 October 2000	TT		GlaxoSmithKline Biologicals S.A.	Hib (PRP)
Menactra	14 January 2005	DT	Sanofi Pasteur, Inc.		Men (A, C, Y and W-135)
MenHibrix	14 June 2012	TT	GlaxoSmithKline Biologicals		Men (C and Y), Hib (PRP)
Menomune-A/C/Y/W-135	9 January 2009		Sanofi Pasteur, Inc.		Men (A, C, Y and W-135)
Menveo	15 March 2010	CRM197		GSK Vaccines S.r.l.	Men (A, C, Y and W-135)
Menveo	19 February 2010	CRM197	Novartis Vaccines and Diagnostics, Inc.		Men (A, C, Y and W-135)
Nimenrix	20 April 2012	TT		Pfizer Limited	Men (A, C, Y and W-135)
PedvaxHIB	27 April 2011	OMPC	Merck & Co, Inc.		Hib (PRP)
Pentacel	20 June 2008	TT	Sanofi Pasteur Limited		Hib (PRP)
Pneumovax 23	6 August 2008		Merck & Co, Inc.		Pneumo (23-valent: 1, 2, 3, 4, 5, 6B, 7F, 8, 9N, 9V, 10A, 11A, 12F, 14, 15B, 17F, 18C, 19F, 19A, 20, 22F, 23F and 33F)
Prevenar	2 February 2001	CRM197		Pfizer Limited	Pneumo (7-valent: 4, 6B, 9V, 14, 18C, 19F, and 23F)
Prevenar 13	9 December 2009	CRM197		Pfizer Limited	Pneumo (13-valent: 1, 3, 4, 5, 6A, 6B, 7F, 9V, 14, 18C, 19A, 19F and 23F)
Prevnar	17 February 2000	CRM197	Wyeth Pharmaceuticals Inc.		Pneumo (7-valent: 4, 6B, 9V, 14, 18C, 19F, and 23F)
Prevnar 13	24 February 2010	CRM197	Wyeth Pharmaceuticals Inc.		Pneumo (13-valent: 1, 3, 4, 5, 6A, 6B, 7F, 9V, 14, 18C, 19A, 19F and 23F)
Synflorix	30 March 2009	PD; TT; DT depending on serotype		GlaxoSmithKline Biologicals S.A.	Pneumo (10-valent: 1, 4, 5, 6B, 7F, 9V, 14 and 23F; 18C; 19F)
TYPHIM Vi	27 March 2014		Sanofi Pasteur, S.A.		Typh (Vi)
Vaxelis	15 February 2016	OMPC		Sanofi Pasteur MSD SNC	Hib (PRP)

Table 1 lists the Food and Drug Administration (FDA) and European Medicines Agency (EMA) approved PS containing vaccines.
